# Experimental Study on Carbonation Durability of Kaolin Strengthened with Slag Portland Cement

**DOI:** 10.3390/ma15031240

**Published:** 2022-02-07

**Authors:** Qingbiao Wang, Yiming Ma, Fuqiang Wang, Zhenyue Shi, Hongyue You, Yuanyuan Tian, Yunfei Liu, Zhongjing Hu, Hongxu Song, Dong Wang, Yueqiang Sun, Rongshuai Yang, Haoran Sun

**Affiliations:** 1 College of Resources, Shandong University of Science and Technology, Tai’an 271019, China; skd990748@sdust.edu.cn (Q.W.);202183300045@sdust.edu.cn (Y.M.); 202083300013@sdust.edu.cn (Y.L.); 202083300021@sdust.edu.cn (Y.S.); 202083300026@sdust.edu.cn (R.Y.); 202083300019@sdust.edu.cn (H.S.); 2 State Key Laboratory of Mining Disaster Prevention and Control Co-Founded by Shandong Province and the Ministry of Science and Technology, Shandong University of Science and Technology, Qingdao 266590, China; 3 National Engineering Laboratory for Coalmine Backfilling Mining, Shandong University of Science and Technology, Tai’an 271019, China; 4 College of Civil Engineering and Architecture, Shandong University of Science and Technology, Qingdao 266590, China; 201983040061@sdust.edu.cn (F.W.); 201982040039@sdust.edu.cn (H.Y.); 201982040032@sdust.edu.cn (Y.T.); 201982040033@sdust.edu.cn (D.W.); 5 College of Safety and Environmental Engineering (College of Safety and Emergency Management), Shandong University of Science and Technology, Qingdao 266590, China; huyang@sdust.edu.cn; 6 College of Energy and Mining Engineering, Shandong University of Science and Technology, Qingdao 266590, China; 201983010016@sdust.edu.cn

**Keywords:** slag Portland cement, carbonation durability, carbonation depth, uniaxial compressive strength test (UCST)

## Abstract

Slag Portland cement is an environmentally friendly and energy-saving product, which is widely used in cement-reinforced soil. This study used slag Portland cement-reinforced soil as the research object and P.O 42.5 + kaolin (POK) as the reference group. The carbonation depth and strength of P.S.A 42.5 + kaolin (PSK) at different curing times were analyzed using carbonation depth, uniaxial ground pressure strength, scanning electron microscopy (SEM), energy-dispersive spectroscopy (EDS) and X-ray diffraction (XRD). The test results show the following: (1) The active substances in PSK samples can react with calcium hydroxide produced during cement hydration and can reduce the content of OH^−^. The PSK samples react with OH^−^ and CO_2_ in the carbonation environment. Both processes considerably reduce the content of OH^−^. (2) Due to the decrease in OH^−^ content, the carbonation durability of slag Portland cement-reinforced soil is significantly less than that of ordinary Portland cement. (3) The carbonation of slag Portland cement-reinforced soil improves its strength. (4) The results of SEM + EDS and XRD confirm the carbonation depth and strength of the POK and PSK samples. The results show that PSK has important applications in subgrade or building grouting materials and in cement-soil mixing piles (walls).

## 1. Introduction

Coastal beach and soft foundation reinforcement is a common engineering problem. Cement soil reinforcement is one of the most commonly used, convenient, and economical methods. However, the manufacture of traditional cement consumes large amounts of energy. With the global promotion of green, carbon neutral development, new requirements are being adopted to reduce cement energy consumption [1]. Slag Portland cement has a beneficial environmental effect because its raw material is an industrial by-product (blast furnace slag). The research on ordinary Portland cement-reinforced soil has progressed significantly, and ordinary Portland cement is widely used as a soil-reinforcement agent.

Kang et al. [2,3] studied the change of unconfined compressive strength of ordinary Portland cement-reinforced soil under different curing times, and measured the effect of curing time on its strength. Pu et al. [4] studied the mechanical properties and micro-properties of ordinary Portland cement-solidified soil. Their tests showed that the unconfined compressive strength of cement-reinforced soil increases with an increase in cement content. Kim et al. [5] obtained the dynamic and static characteristics of ordinary Portland cement-reinforced soil through unconfined compression testing; they demonstrated that the unconfined compressive strength of ordinary Portland cement-reinforced soil increases with time. Yao et al. [6] studied the effect of curing time of ordinary Portland cement-reinforced soil on unconfined compressive strength, and concluded that the strength increased with increased curing time. Ding et al. [7] studied the effect of consolidation time on the unconfined compressive strength of soil strengthened with ordinary Portland cement. Zhou et al. [8] carbonized ordinary Portland cement-reinforced soil. The results show that carbonation reduces the strength of ordinary Portland cement-reinforced soil. Shah et al. [9] showed that the volume of all cement shrinks after carbonation. Liu et al. [10] observed through X-ray diffraction (XRD) tests that the main polycrystalline form of carbonated ordinary Portland cement is calcite (CaCO_3_).

In addition to the voluminous research on ordinary Portland cement, the research on slag Portland cement is becoming more and more mature. Luo et al. [11] studied the influence of curing temperature on the compaction carbonation behavior of steel slag. Sanjuán et al. [12] observed the high carbonation rate of abrasive slag cement, and proposed a calculation model of carbonation depth and CO_2_ diffusion coefficient in ground granulated blast-furnace slag mortars as a function of the curing period and the amount of ground granulated blast-furnace slag. Sanjuán et al. [13] studied the carbonation mechanism of calcium hydroxide (CH) and hydrogenated calcium silicate (C-S-H) from the aspects of microstructure and moisture characteristics. Andrade and Sanjuán [14] studied the hydration and fixation of carbon dioxide through a large number of experiments and described the factors that affect the efficiency of carbon dioxide fixation. Sanjuán [15] studied the effect of carbon dioxide absorption by blast-furnace-slag mortar on curing strength. Sanjuán et al. [16] also analyzed the advantages and disadvantages of the ground granulated blast-furnace slag efficiency coefficient and gave their own empirical choices. Pu et al. [17] studied the carbonation resistance of slag concrete and found that the strength of slag concrete did not decrease significantly after complete carbonation. Wu et al. [18] employed XRD and scanning electron microscopy (SEM) to examine the micro-morphology of solidified soil and obtain the mineralogical differences with erosion time. Monsif et al. [19] found that the strength of slag Portland cement-reinforced soil increased with time. Allahverdi et al. [20] studied the chemical activity of slag after adding ordinary Portland cement to it. The results showed that the chemical activity was significantly improved by adding slag to cement. Ge et al. [21] studied the effect of slag Portland cement on soil strength and concluded that the unconfined compressive strength increased with the increase of the ratio of slag cement to soil. Liu et al. [22] studied the strength characteristics of slag Portland cement-reinforced soil under different curing times; their research showed that slag Portland cement-reinforced soil can effectively improve soil strength. Saafan et al. [23] studied the microstructure, physicochemical properties, mechanical properties, and durability of slag Portland cement mortar.

Although many scholars have performed significant research on slag Portland cement-reinforced soil, prior research on cement-reinforced soil has focused primarily on the strength and carbonation of ordinary Portland cement-reinforced soil, whereas the research on slag Portland cement has mainly focused on the mechanical and carbonation properties of pure mortar samples or concrete. The research on slag Portland cement-reinforced soil is relatively sparse, focusing mainly on its mechanical properties, and there has been little research on the carbonation properties of slag Portland cement-reinforced soil. Carbonation performance is an important index of cement-reinforced soil. In order to understand whether slag Portland cement can replace ordinary Portland cement for soil reinforcement, it is necessary to study its carbonation performance.

In this work, the carbonation durability of slag Portland cement-reinforced soil and that of ordinary Portland cement-reinforced soil were studied, analyzed, and compared through a combination of macro tests and micro tests. In the macro test, the carbonation behaviors of slag Portland cement-reinforced soil and ordinary Portland cement-reinforced soil were studied through carbonation depth testing and unconfined compression testing. Through XRD and SEM tests, the microstructure characteristics, pore distribution, degree of particle cementation, and hydration product morphology of reinforced soil samples were analyzed; and subsequently the hydration mechanisms and micro characteristics of hydration reaction products of reinforced soil before and after carbonation were analyzed. Combining the results obtained from the macro tests and the micro tests, it can be concluded that slag Portland cement-reinforced soil is easier to carbonize than ordinary Portland cement-reinforced soil, and that carbonation increases the strength of the cement-soil. Therefore, slag Portland cement can be used for the reinforcement and improvement of soil having poor engineering characteristics.

## 2. Materials and Methods

### 2.1. Experimental Materials and Sample Preparation

In order to test the reinforcement effect of slag cement on soil, ordinary Portland cement was designated as the control substance. The main experimental materials were kaolin, P.O 42.5 ordinary Portland cement and P.S.A 42.5 slag Portland cement. The kaolin was produced by Shanghai Jiujie Industrial Co., Ltd (Shanghai, China).; the P.O 42.5 ordinary Portland cement was produced by Shandong Weizhou Cement Technology Co., Ltd. (Weifang, China); and the P.S.A 42.5 slag Portland cement was produced by Sinoma Zhuzhou Cement Co., Ltd (Zhuzhou, China). Table 1 [24] shows the specific parameters.

The test materials were analyzed by XRD. According to the XRD pattern, the main composition of kaolin is Al[Si_4_O_10_](OH)_8_. The main components of P.O 42.5 and P.S.A 42.5 are C3S, SiO_2_, CaSO_4_. This corresponds to the main chemical composition of each test material in Table 1.

The P.O 42.5 or P.S.A 42.5 cement was mixed with kaolin at a mass ratio of 10:3 [25], and was used to fabricate two types of samples: Sample 1 was a 100 mm cube, and Sample 2 was a cylindrical test block with a radius of 50 mm and a height of 100 mm. Samples 1 and 2 were fabricated from each of two different sample materials: P.O 42.5 + kaolin (POK) and P.S.A 42.5 + kaolin (PSK). Depending on the desired curing time and processing sequence, the prepared samples needed standard curing first, and some needed an additional carbonation curing step. The standard curing temperature was 20 °C at a relative humidity of 95%; the carbonation curing temperature was 20 °C at a relative humidity of 70%; and the CO_2_ concentration was maintained at 20% [26,27]. Sample preparation is shown in Table 2.

### 2.2. Experimental Scheme

The tests were carried out after the sample had been cured for the specified time. The phase I tests were performed first: a carbonation test for sample 1 and a uniaxial compressive strength test for sample 2. During the phase II test, a middle fragment of sample 2 that had been damaged by the compressive testing was used for the XRD and SEM tests.

#### 2.2.1. Carbonation Test (CDT)

After 28 days of standard curing of sample 1, carbonation curing was carried out. The samples cured for 3, 7, 14, and 28 days, were used for the carbonation test. The test block was cut along the center line of the upper surface, and 1% phenolphthalein ethanol solution was sprayed on the section; in this test, the non-carbonized area shows red, the carbonized area does not change color, and the height of the red area is the carbonation depth. At least 5 measuring points were taken for each test piece, and the average depth of the measuring points was defined as the carbonation depth. Three samples were used for each curing time, and the average carbonation depth of the three samples was taken as the carbonation depth at that curing time.

#### 2.2.2. Uniaxial Compressive Strength Test (CUST)

The uniaxial compressive strength test was performed after sample 2 reached its curing time. The uniaxial compressive strength test was divided into four test groups: (1) a POK sample that had been cured for 56 days (standard curing for 56 days, abbreviated as S56); (2) a POK sample that had been carbonized for 28 days after standard curing (carbonation curing for 28 days, abbreviated as C28); (3) a PSK sample that had received standard curing for 56 days (S56); (4) a PSK sample that had received standard curing for 28 days and was then carbonized for 28 days (S28 + C28). A microcomputer controlled electro-hydraulic servo universal testing machine was used to test the samples. The loading rate was 0.5 MPa/s, and the average value of three samples in each group was taken as the uniaxial compressive strength of this group.

#### 2.2.3. XRD and SEM + EDS Tests

**Figure 1:**
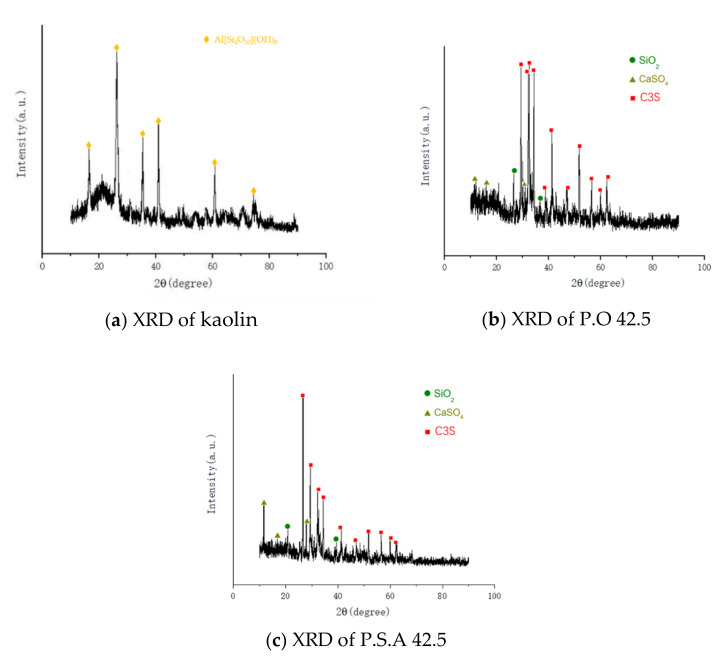
XRD of raw materials.

The samples used in the XRD and SEM + EDS tests were the fragments of sample 2 after its failure during the uniaxial compressive strength test, which were then ground into powder to be used as test samples for the current test. A DX-2700 X-ray diffractometer was used for the XRD, and a Quanta250 scanning electron microscope was used for the SEM + EDS tests. These two pieces of equipment, shown in Figure 1, are located at the National Engineering Laboratory of Shandong University of science and technology.

## 3. Results

In accordance with the experimental group design in Table 2, the carbonation test, uniaxial ground pressure strength test, SEM + EDS test, and XRD test were carried out for POK and PSK sample materials; and the collected test data were processed and analyzed. The specific test results will be described in detail in the following subsections.

### 3.1. Analysis of Carbonation Depth Test (CDT) Results

**Figure 2:**
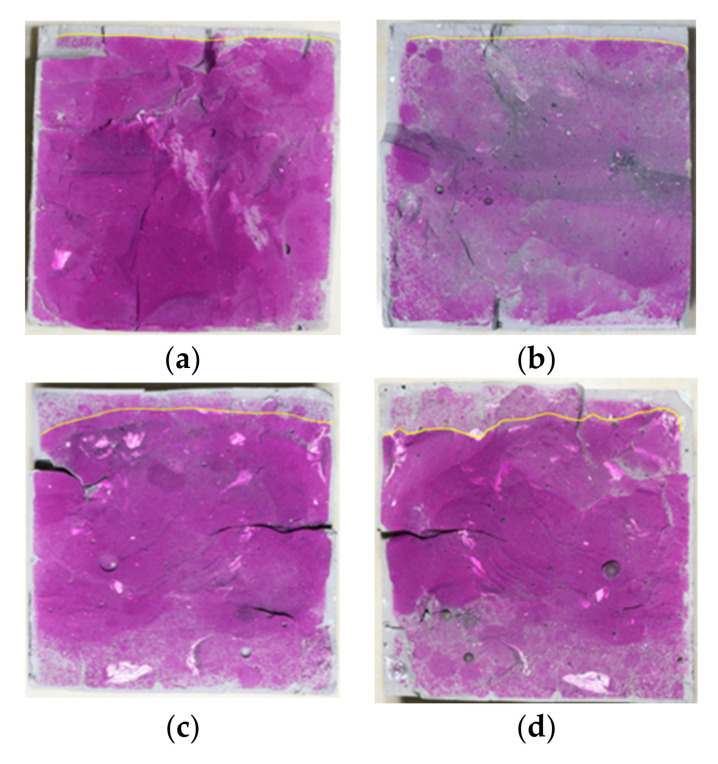
Phenolphthalein test diagram of carbonation depth in POK samples that had been subjected to differing amounts of carbonation curing—(**a**) 3 d (**b**) 7 d (**c**) 14 d and (**d**) 28 d.

**Figure 3:**
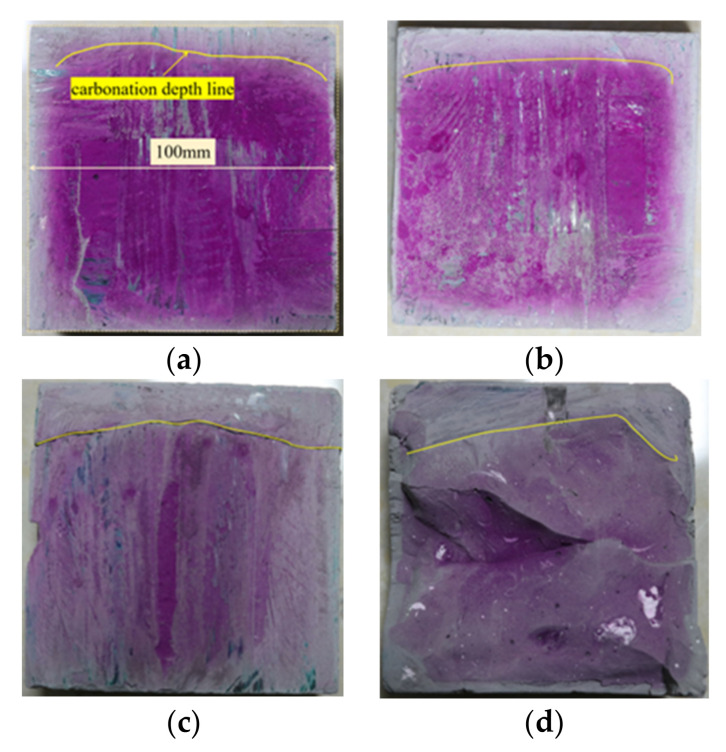
Phenolphthalein test diagram of carbonation depth in PSK samples that had been subjected to differing amounts of carbonation curing—(**a**) 3 d (**b**) 7 d (**c**) 14 d and (**d**) 28 d.

The carbonation depths of POK and PSK samples after 3, 7, 14, and 28 days of carbonation curing were tested using a phenolphthalein reagent. The test results are shown in Figure 2 and Figure 3. The phenolphthalein reagent used was a weak acidic solution that is colorless in acidic solutions and turns red in alkaline solutions. The greater the OH^−^ concentration of the solution, the darker the color of the reagent. It can be seen from these two groups of pictures that for the same group of samples, the carbonation depth also deepens with increasing curing time. This is because a large amount of OH^−^ is produced in the hydration process of cement, and the sample is alkaline at the initial stage of preparation. However, CO_2_ in the carbonation curing environment continuously penetrates into the sample from outside and reacts with OH^−^ in the carbonation process, which greatly reduces the content of OH^−^; therefore, the carbonation depth increases with age. At the same time, the red color of the phenolphthalein reagent on POK is significantly deeper than that on PSK, and the red color of PSK becomes lighter and lighter with the increase of age. According to the published literature [28,29], slag cement contains a number of active substances such as SiO_2_, Al_2_O_3_, and Fe_2_O_3_ that can also react with OH^−^ and reduce the content of OH^−^ in the sample even further, resulting in the light color seen in the phenolphthalein tests of PSK.

**Figure 4:**
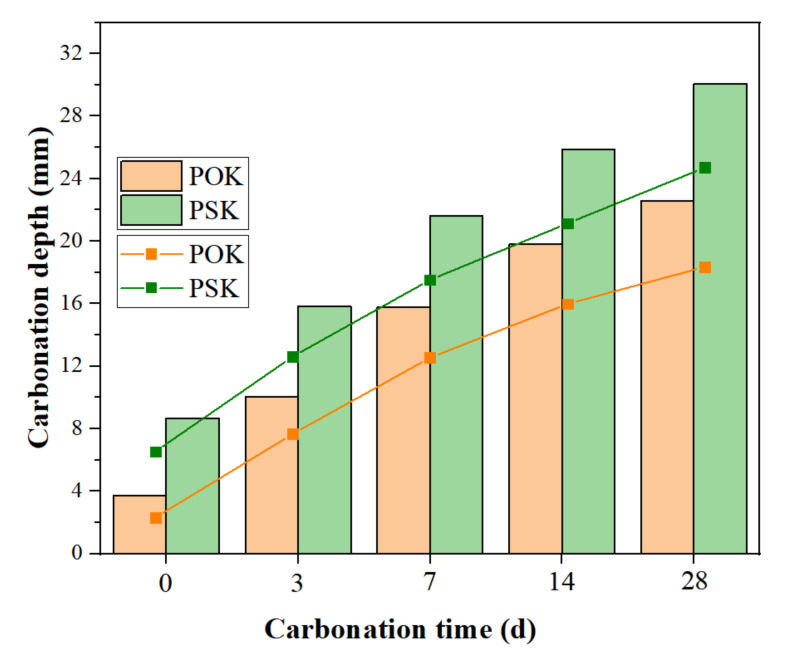
Relationship between carbonation depth and curing time of sample.

As can be seen from Figure 4, the carbonation depth of POK increases from the initial 3.7 mm (0 d) to 22.6 mm (28 d), and the carbonation depth of PSK increases from the initial 8.7 mm (0 d) to 30.1 mm (28 d). The carbonation depth of both materials increased with increasing curing time, but the rate of increase gradually slowed down for both the materials. Moreover, the carbonation depth of PSK is significantly higher than that of POK at any given time. The reason for this phenomenon is similar to the principle of color depth of phenolphthalein. It is because CO_2_ and the active substances in slag cement react with OH^−^, which increases the carbonation effect of PSK and makes its carbonation depth significantly higher than that of POK. Therefore, the carbonation durability of slag cement-reinforced soil is significantly weaker than that of ordinary Portland cement. Shi et al. [30], Bakharev et al. [31], Puertas et al. [32], and Pacheco-Torgal [33] also found that slag cement is more prone to carbonation.

### 3.2. Analysis of CUST Results

**Figure 5:**
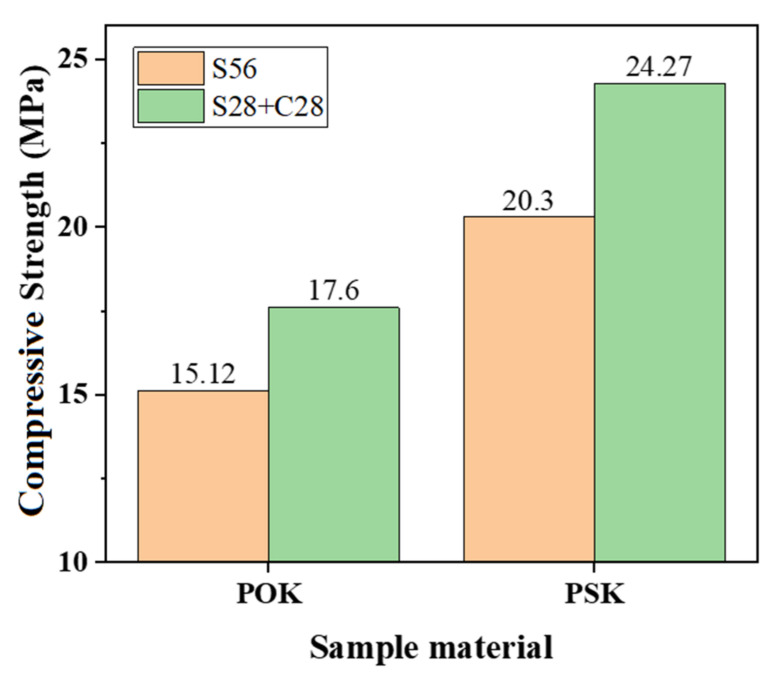
Uniaxial compressive strength of POK and PSK samples after S56 and S28 + C28 aging.

Observe Figure 5, the uniaxial compressive strength of the same sample is different under different curing conditions. Specifically, put POK under the standard curing for 28 days and under the carbonation curing for 28 days (S28 + C28), its uniaxial compressive strength is 17.6MPa. Then, put POK under the standard curing for 56 days(S56), its uniaxial compressive strength is 15.12MPa. The uniaxial compressive strength of POK under carbonation curing (S28 + C28) is 16.4% higher than the uniaxial compressive strength of POK without carbonation curing condition. Similarly, put PSK under the standard curing for 28 days and under the carbonation curing for 28 days (S28 + C28), its uniaxial compressive strength is 24.27MPa. Then, put PSK under the standard curing for 56 days (S56), its uniaxial compressive strength is 20.3 MPa. The uniaxial compressive strength of PSK under carbonation curing (S28 + C28) is 19.56% higher than the uniaxial compressive strength of PSK without carbonation curing condition. In general, the uniaxial compressive strength of the sample was improved by the curing method of standard curing for 28 days and carbonation curing for 28 days (S28 + C28). In other words, carbonation curing enhances uniaxial compressive strength. Therefore, carbonation is beneficial to improve the strength of concretes [34,35].

It can also be observed in Figure 5 that the uniaxial compressive strength of different samples is different even though samples are under the same curing conditions. Specifically, put PSK and POK under the standard curing for 56 days (S56), the uniaxial compressive strength of PSK is 20.3 MPa, but the uniaxial compressive strength of POK is 15.12 MPa. The strength of PSK is 34.26% higher than the strength of POK. In the same way, put PSK and POK under the standard curing for 28 days and under the carbonation curing for 28 days (S28 + C28), the uniaxial compressive strength of PSK is 24.27 MPa, but the uniaxial compressive strength of POK is 17.6 MPa. The strength of PSK is 37.9% higher than the strength of POK. In general, the uniaxial compressive strength of PSK is obviously greater than POK, when they are under different curing conditions. Susan A. Bernal [36] also reached a similar conclusion.

There are two reasons for the phenomenon: (1) CH produced in the hydration reaction is harmful to strength. In the carbonation curing process, due to the high concentration of CO_2_ in the environment, carbonation is more sufficient, then the content of CH was decreased greatly, and the main precipitate that is CaCO_3_ increased [37]. (2) Active substances such as SiO_2_, Al_2_O_3_ and Fe_2_O_3_ contained in slag cement react with CO_2_ in carbonation curing environment to form CaSiO_3_, CaO · Al_2_O_3_ · 6H_2_O and other substances. Both reactions consume OH^−^ produced in the process of cement hydration, which is unfavorable for reinforced concrete and may lead to reinforcement corrosion. However, for the reinforced soil structure without steel reinforcement, the generated solid matter increases the strength of the sample. Therefore, after carbonation curing, the strength of the cement reinforced soil sample is improved.

### 3.3. Analysis of SEM and EDS Data

Through the SEM + EDS method, combined with the cement hydration and carbonation results, as well as using XRD technology, we can roughly judge the products created during cement hydration and carbonation, and determine the main products.

**Figure 6:**
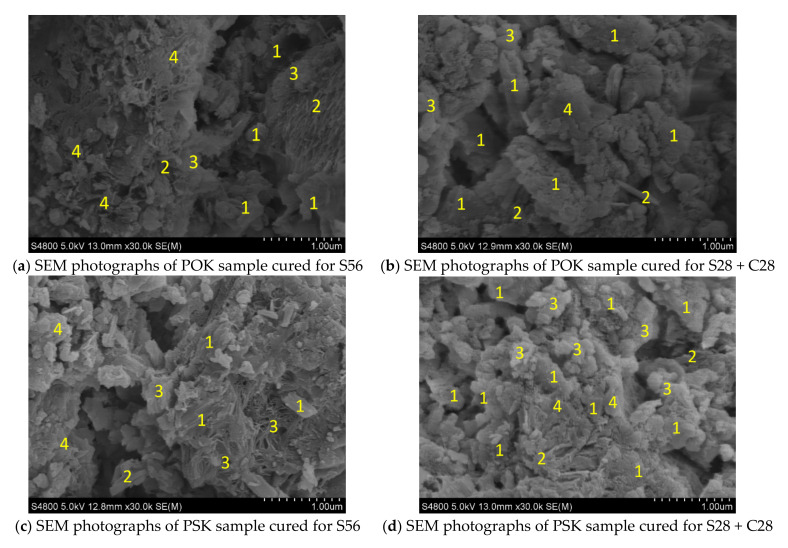
SEM photographs of samples. The numbers in the figures denote: 1. Calcium carbonate (CaCO_3_). 2. Ettringite (AFt). 3. Hydrogenated calcium silicate (C-S-H). 4. Calcium hydroxide (CH).

Different substances formed by cement hydration and carbonation have their unique shapes under SEM observation. It can be seen from Figure 6a–d that after carbonation of the POK and PSK samples, the amount of calcium carbonate (CaCO_3_) increases significantly, mainly in the form of sheet overlap, particle agglomeration, and blocks. Ettringite or alumina/ferric oxide/trisulfate (AFt) is needle-shaped or rod-shaped and becomes thicker after carbonation. Before and after carbonation, hydrogenated calcium silicate (C-S-H) is abundant, mainly in cloud clusters and clusters. The amount of CH (mainly present as hexagonal flakes) decreases significantly [38]. At the same time, the sample structure is more compact after carbonation, which mainly leads to the increase in sample strength. By comparing the POK sample in Figure 6b with the PSK sample in Figure 6d, it can be seen that after 28 days of carbonation, the CaCO_3_ blocks in the POK sample are dense, and the needle shape of AFt in the PSK sample is significantly increased and coarsened, which is due to the partial formation of C-S-H, AFt, etc., by the active substances in the PSK sample under the action of the hydration reactions [39]. The average content of C-S-H is high, but the boundary of the C-S-H cloud cluster in the PSK sample is clearer and is arranged more densely [27]. After carbonation of POK and PSK, CH was mostly consumed and therefore, decreased significantly.

The hydration process of cement produces a large amount of CH, but the carbonation process consumes CH, and the active substances in PSK also react with CH, increasing the contents of CaCO_3_, C-S-H and AFt. Thus, the strength of the sample is improved. The large consumption of CH leads to a reduction in the alkalinity of the sample, which is confirmed by the light discoloration of the PSK sample in Section 3.1 and the strength analysis results in Section 3.2.

**Figure 7:**
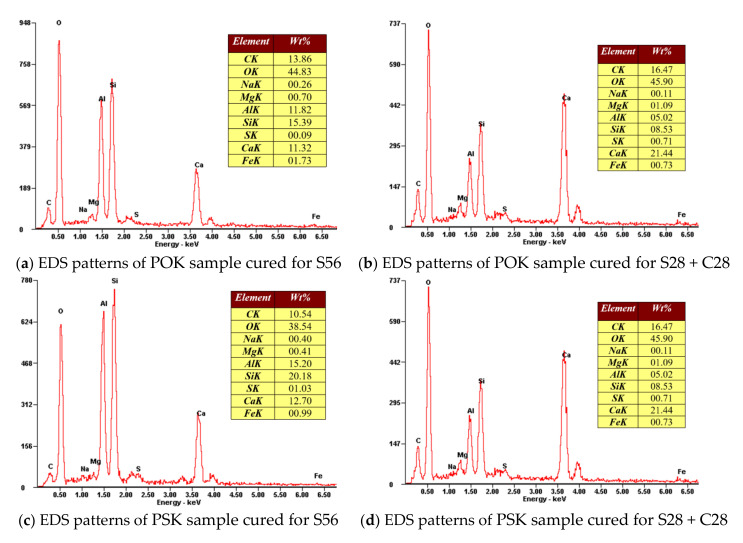
EDS patterns of various samples.

The EDS spectra were measured using SEM. It can be seen from Figure 7 that the main elements in all the samples are O, C, Ca, Si, and Al; and the samples also contain small amounts of Mg, Na, S, and Fe. It can be seen by comparing Figure 7a–d that the mass ratio of C to O increases significantly after carbonation in both the POK sample and the PSK sample. This is caused by the sample absorbing CO_2_ in the carbonation environment. It can be seen from Figure 7b,d that after carbonation of the PSK sample, the content of C and O increases compared with standard curing conditions, but the increase is less than that of the POK sample in the carbonation environment. This is because the active substances in the PSK sample react with CH while carbonizing. To some extent, this reduces the ability of the PSK to be carbonized by CO_2_ and therefore, the amount absorbed is relatively less.

### 3.4. Analysis of XRD Test Results

**Figure 8:**
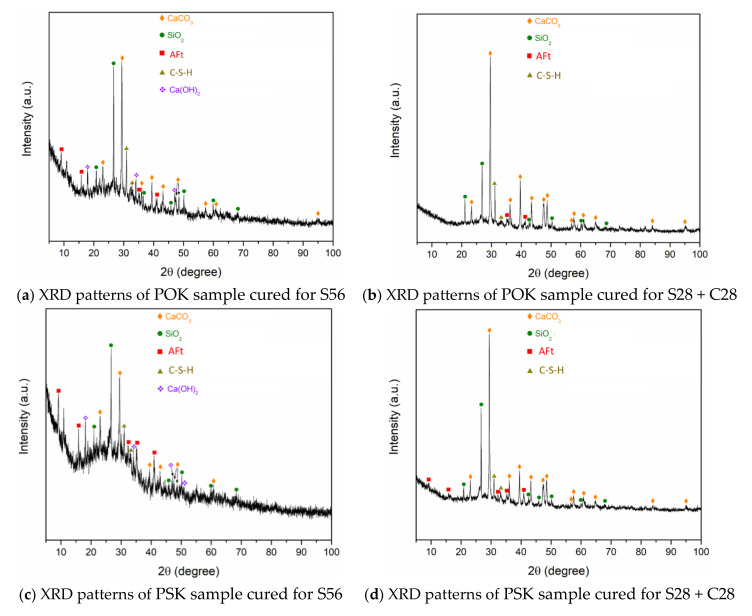
XRD patterns of samples.

It can be seen from Figure 8 that the products of POK and PSK samples in the standard curing and carbonation environments are the same, and the main products after hydration and carbonation are CaCO_3_, CaSO_4_, SiO_2_ and Aft. By combining SEM + EDS analysis and hydration–carbonation reaction analysis, there are other products such as CH, C-S-H, AFm, C2S, C_3_AH_6_, etc. [40,41,42].

By comparing Figure 8b with Figure 8a, and Figure 8d with Figure 8c, it can be seen that after carbonation of the POK and PSK samples, the amount of CaCO_3_ increased significantly and that the increase was greater in the POK. This is because the carbonation reaction is more sufficient. More CH, Aft, AFm and CO_2_ produced CaCO_3_ and other products [43,44]. The reaction formulas are shown in Equations (1)–(3). The strength of the sample increases with the increase of CaCO_3_ content and the decrease of CH content, this is consistent with the conclusion in Section 3.2.

Ca(OH)_2_ + H_2_CO_3_→CaCO_3_ + 2H_2_O (1)

3CaO·Al_2_O_3_·3CaSO_4_·32H_2_O + 3CO_2_→3CaCO_3_ + 3CaSO_4_ + Al_2_O_3_·xH_2_O + (32 − x)H_2_O (2)

3(3CaO·Al_2_O_3_·CaSO_4_·12H_2_O) +9CO_2_→9CaCO_3_ + 3CaSO_4_ + 3Al_2_O_3_·xH_2_O + (32 − 3x)H_2_O (3)

By comparing Figure 8b with Figure 8d, it can be seen that after carbonation of the POK and PSK samples, the amount of CaCO_3_ in PSK is significantly higher than that in POK. This is because that CH, one of the hydration products in PSK sample, reacts with active substance in PSK sample to produce C-S-H, etc. [45,46]. Thus, the strength of the sample increased. This is consistent with the conclusion in Section 3.2. At the same time, the active substances contained in the PSK samples react preferentially with CH, thus competing for CH that should have been carbonized by CO_2_. Therefore, the CO_2_ absorbed by the PSK sample is less than that of the POK sample, and the content of CaCO_3_ in the POK sample is relatively high. This is consistent with the CO_2_ content described in Section 3.3.

## 4. Discussion and Suggestions

The results of this study show that slag Portland cement is not suitable for reinforced concrete because of its high carbonation characteristics that can easily lead to reinforcement corrosion. However, its strength effectively improves after carbonation. Therefore, it can be widely used in foundation reinforcement, such as for the prevention of uneven settlement of subgrade or buildings, or as grouting material. Moreover, it can also be used in the construction of cement–soil mixing piles and walls.

## 5. Conclusions

By testing POK and PSK samples after curing in the S56 and S28 + C28 environments, the carbonation depth and sample strength were studied. The micro-morphology and elemental composition were tested by SEM + EDS, and the products of hydration and carbonation were analyzed by XRD. The specific conclusions are as follows:

(1) The content of OH^−^ was reduced during hydration and carbonation of the PSK samples. The active substances contained in PSK samples can react with the CH produced during cement hydration to reduce the content of OH^−^. In the S28 + C28 curing environment, OH^−^ in the PSK samples reacts with CO_2_. Both processes greatly reduce the content of OH^−^.
(2) The POK and PSK samples were carbonized in the S28 + C28 environment. The carbonation depth in the PSK sample at each curing time was deeper than that of the POK sample, and the color of the phenolphthalein reagent on PSK was significantly lighter than in the case of the POK sample. This shows that the carbonation durability of slag Portland cement is weaker than that of ordinary Portland cement.
(3) The strength of the PSK sample after carbonation was higher than that without carbonation. The strength of the PSK sample after carbonation was higher than that of the POK sample after carbonation. This shows that the carbonation of slag Portland cement-reinforced soil improves its strength.
(4) After comprehensive analysis of SEM, EDS and XRD, the elements and products of POK and PSK samples before and after hydration and carbonation are basically the same. The main products after hydration and carbonation are CaCO_3_, CaSO_4_, SiO_2_, Aft, AFm, CH, C-S-H, C2S, etc. The increase of crystals content such as calcium carbonate and the decrease of calcium hydroxide content are the main factors for the improvement of PSK sample strength.

## Figures and Tables

**Table 1:** Basic chemical properties of the experimental materials.

Material Type	CaO (%)	SiO_2_ (%)	Al_2_O_3_ (%)	SO_3_ (%)	Fe_2_O_3_ (%)	MgO (%)	NaO_2_ (%)	K_2_O (%)	Cl (%)	LOI (%)	Specific Surface Area (m^2^/kg)
Kaolin	0.55	57.03	30.32	0.07	1.42	0.49	/	/	/	/	/
P.S.A 42.5	51.81	26.48	10.09	2.54	2.58	3.96	/	/	/	1.33	326
P.O 42.5	56.23	25.09	6.02	2.13	3.87	2.38	0.46	0.59	0.05	2.55	341

**Table 2:** Sample preparation and test type.

Sample Type	Sample Material	Curing Sequence (Time and Sequence)				Test Sequence		
		a. Standard Curing		b. Carbonation Curing		a. Phase I Test	b. Phase II Test	
		Time	Quantity	Time	Quantity	Test Type	Test Type	
Sample 1	P.O 42.5 + Kaolin (POK)	28	12	3	3	CDT	/	
				7	3			
				14	3			
				28	3			
	P.S.A 42.5 + Kaolin (PSK)	28	12	3	3			
				7	3			
				14	3			
				28	3			
Sample 2	P.O 42.5 + Kaolin (POK)	56	3	/	/	CUST	XRD	SEM
		28	3	28	3		XRD	SEM
	P.S.A 42.5 + Kaolin (PSK)	56	3	/	/		XRD	SEM
		28	3	28	3		XRD	SEM

Remarks: CDT is the carbonation depth test. CUST is the uniaxial compressive strength test.

## Data Availability

The data presented in this study are available on request from the corresponding author.
